# Supplementary material for: Face cells encode object parts more than facial configuration of illusory faces
Source: Nat Commun. 2024 Nov 14;15:9879. doi: 10.1038/s41467-024-54323-w (PMC11564726; doi:10.1038/s41467-024-54323-w)
Supplement: Supplementary file 1 — Supplementary Information [file 41467_2024_54323_MOESM1_ESM.pdf]

Supplementary Information

Face cells encode object parts more than facial configuration of illusory faces.

Saloni Sharma[1*], Kasper Vinken[1], Akshay V. Jagadeesh[1], Margaret S. Livingstone[1]

[1]Department of Neurobiology, Harvard Medical School, Boston, MA 02115, USA

[*]Corresponding author: Saloni_Sharma@hms.harvard.edu

This document contains 8 supplementary figures and 2 supplementary tables:

Supplementary Fig. 1. Pareidolia selectivity for scrambled images is not driven by image familiarity.

Supplementary Fig. 2. Scrambling-induced spurious configuration negatively affects pareidolia selectivity.

Supplementary Fig. 3. Quadrant scrambling does not eliminate face selectivity of face cells.

Supplementary Fig. 4. Quadrant scrambling does not eliminate the negative correlation between face selectivity and object rank correlation.

Supplementary Fig. 5. Face deprived and normal monkeys do not differ in face selectivity.

Supplementary Fig. 6. Pareidolia selectivity of neural units in face deprived monkeys falls within the range of normal monkeys.

Supplementary Fig. 7. Pareidolia selectivity is predicted by the feature-tuning estimated from non-face objects.

Supplementary Fig. 8. The contribution of isolated quadrants in driving the face-cell pareidolia selectivity per monkey.

Supplementary Table 1. Correlation between pareidolia and face selectivity per monkey.

Supplementary Table 2. Correlation between pareidolia selectivity for eye parts only and full pareidolia images.

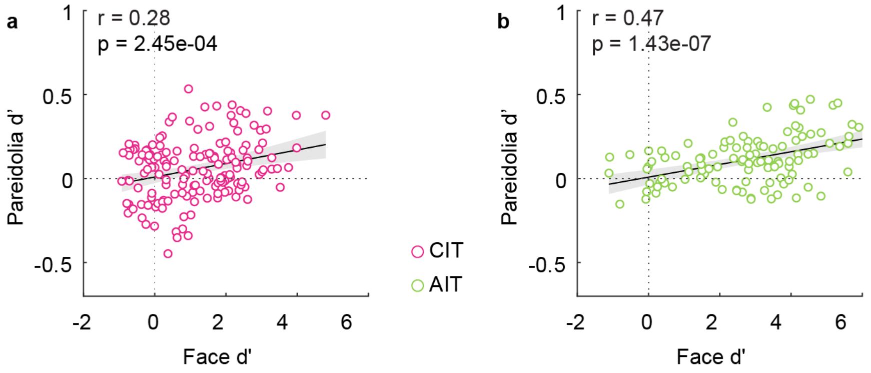

Supplementary Fig. 1. Pareidolia selectivity for scrambled images is not driven by image familiarity. a. Scatterplot showing the correlation between face selectivity (face d') plotted on the x-axis and pareidolia selectivity (pareidolia d') on the y-axis. For this experiment, the monkeys only saw quadrant scrambled versions of unfamiliar pareidolia images, without seeing the original versions. Each dot depicts a neural unit in CIT (n = 162). The black line indicates an ordinary least squares (OLS) linear regression fit, with shaded 95% confidence intervals bands. The values on the top left corner depict the Pearson's correlation (r) and the corresponding p-value calculated using the corr function in MATLAB (R2021b). b. Scatterplot shows the correlation between face and pareidolia selectivity in AIT (n = 114). Same conventions as in a. We found that even for unfamiliar scrambled images, face units in CIT and AIT were pareidolia selective (CIT = 0.1 ± 0.16, t_{85} = 5.79, p = 1.14 x 10^{-7}, AIT = 0.14 ± 0.15, t_{93} = 8.78, p = 7.72 x 10^{-14}), with a positive correlation between face and pareidolia selectivity (CIT: n = 162, Pearson's r = 0.28, p = 2.54 x 10^{-4}, AIT: n = 114, r = 0.47, p = 1.43 x 10^{-7}).

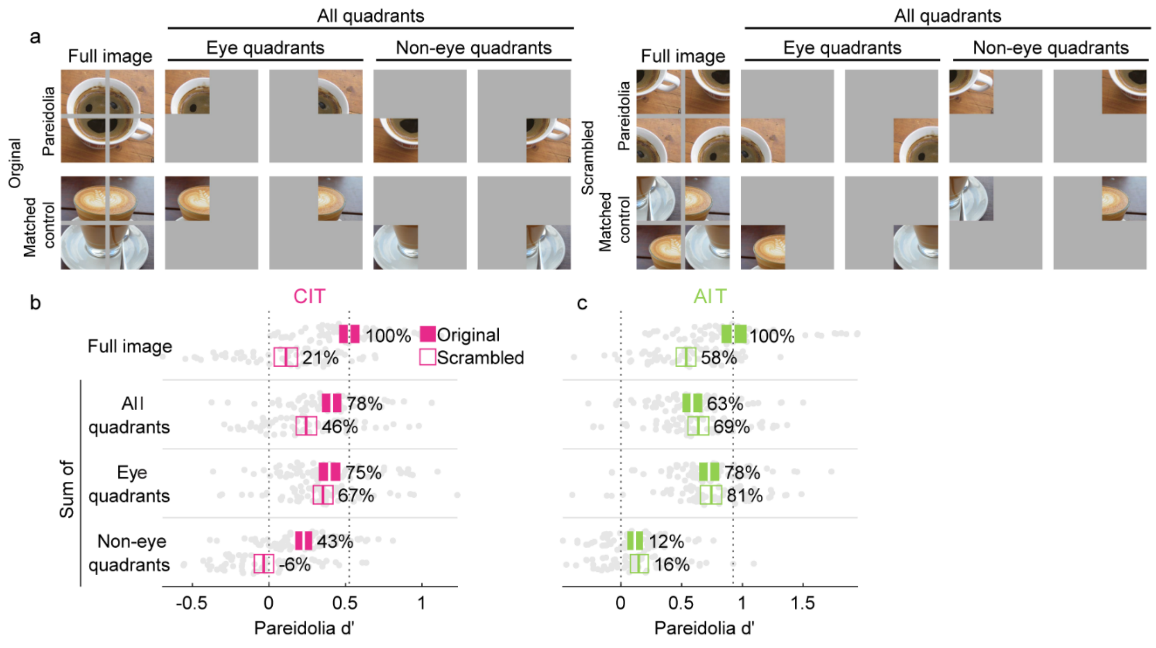

Supplementary Fig. 2. Scrambling-induced spurious configuration negatively affects pareidolia selectivity. a. An example original and scrambled pareidolia and matched control image, and the quadrants that were presented in isolation. A gray cross was introduced in the full original image to ensure the shared presence of quadrant edges in both original and scrambled images. The gray background in the single-quadrant images was the same color as the screen background, which made it appear as if the quadrants were

presented in isolation. The quadrants were divided into “eye” and “non-eye” categories to investigate their contribution to pareidolia selectivity of face-cells. b. The pareidolia d’ in CIT for face units (face d’>1; n = 69) for full images, the average d’ for the sum of all quadrants, the average d’ for the sum of only the eye quadrants and the average d’ for the sum of only the non-eye quadrants for original and scrambled images. Colored boxes indicate 95% bootstrapped CI, calculated by resampling sites, with the central vertical line indicating the mean d’ across units. The numbers adjacent to the colored boxes indicate the percentage of the mean d’ value relative to the d’ of the full original images (shown in Fig. 6). Beeswarm charts show the d’ of each neural unit, where each grey circle represents a neural unit. c. Same conventions as in (b), except for face units in AIT (n = 65). We repeat the results shown in Fig. 6 here for easy comparison between original and scrambled images. We first compared the pareidolia d’ for the original images to the average pareidolia d’ for the scrambled full images and for the sum of the isolated quadrants. In CIT, the average pareidolia selectivity based on scrambled full images was only 21% of the average for the original full images (t_{68} = 11.2, p = 4.63 x 10^{-17}). However, the average pareidolia selectivity of the summed response of these four quadrants in central IT was 78%, higher than the 21% for the scrambled full images. On the other hand, the average pareidolia selectivity based on the sum of the response to isolated quadrants presented in the scrambled positions was 46%. Thus, changing the position of the object parts to the scrambled image locations further reduced pareidolia selectivity by 32% (78% - 46%; t_{68} = 5.13, p = 2.62 x 10^{-6}). Further, the average pareidolia selectivity for individual quadrants in scrambled locations was still twice as high as the pareidolia selectivity for the full scrambled images (46% - 21%; t = -3.8, p = 2.68 x 10^{-4}), suggesting a *negative* effect of the scrambled configuration on pareidolia selectivity. Taken together, these results suggest that quadrant scrambling reduced pareidolia selectivity in central IT for three reasons: because it moved the parts to a new position, eliminated the face-like configuration, and introduced a new configuration. In AIT, scrambling the configuration significantly reduced the average pareidolia selectivity (t_{64} = 9.92, p = 1.46 x 10^{-14}) to 58% of the average for the full original images, suggesting that the impact of scrambling is less profound than in CIT. This value was comparable to the average pareidolia selectivity values based on the sum of the four quadrants in the original positions (63%; Fig. 6c). Further, there was no major negative effect of the scrambled configuration, i.e., when the quadrants took on new positions (63% - 58%; t = 1.7, p = 0.095). Consistent with this, the pareidolia selectivity based on the sum of quadrants in the scrambled positions was comparable (69%), suggesting that the absolute position of the scrambled quadrants did not reduce pareidolia selectivity. Overall, the result that pareidolia selectivity was lowest for scrambled images (compared to summing across all four individual quadrants) points to a negative effect of the spurious configuration introduced by scrambling, particularly in CIT. Example images shown in panel a. adapted from Wardle, S.G., Taubert, J., Teichmann, L. et al. Rapid and dynamic processing of face pareidolia in the human brain. Nat Commun 11, 4518 (2020). https://doi.org/10.1038/s41467-020-18325-8 released under a CC BY license: https://creativecommons.org/licenses/by/4.0/.

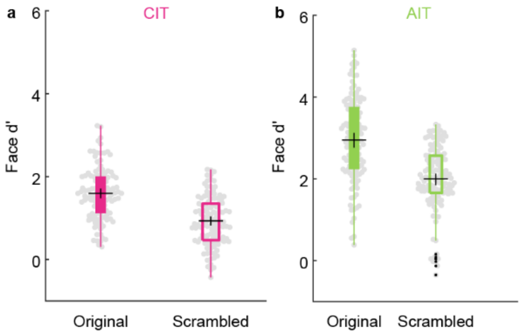

Supplementary Fig. 3. Quadrant scrambling does not eliminate face selectivity of face cells. a. Average face selectivity shown in box plots for face units (face d’>1) in CIT (113/208) for original (filled boxplot) and scrambled (open boxplot) faces presented in Experiment 1. The black central horizontal line shows the mean response, the black central vertical line indicates confidence intervals, and the bottom and top edges of the box depict the 25th and 75th percentiles, respectively. Whiskers (vertical lines in color) show the most extreme data points not considered outliers. Beeswarm plots show the face selectivity of each individual unit. b.

Average face selectivity for face units (face d'>1) in AIT (122/163). Same conventions as in a. Original face d' shown in a and b represents the selectivity for real faces (32 human + 68 monkey) vs 100 nonpareidolia control objects and scrambled face d' represents the selectivity for 100 real scrambled faces vs 100 scrambled nonpareidolia control objects. Scrambling real faces reduced but did not eliminate face selectivity in either of the two regions (CIT: original = 1.6, scrambled = 0.93, t_{112} = 22.03, p = 1.6 x 10^{-42}, AIT: original = 2.95, scrambled = 1.99, t_{121} = 20.98, p = 3.94 x 10^{-42}).

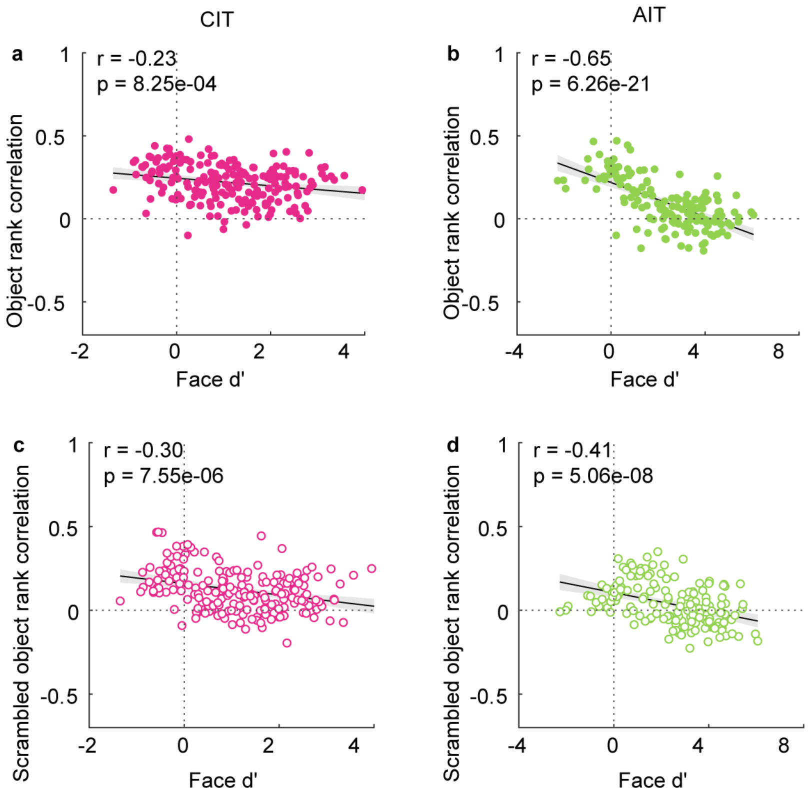

Supplementary Fig. 4. Quadrant scrambling does not eliminate the negative correlation between face selectivity and object rank correlation. a. Scatterplot showing the correlation between face selectivity (face d') plotted on the x-axis and object rank correlation on the y-axis. Each dot depicts a neural unit in CIT (n = 208, pooled across 4 monkeys). The black line indicates an ordinary least squares (OLS) linear regression fit, with shaded 95% confidence intervals bands. The values on the top left corner depict the Pearson's correlation (r) and the corresponding p-value calculated using the corr function in MATLAB (R2021b). b. Scatterplot showing the correlation between face and object rank correlation in AIT (n = 163, pooled across 5 monkeys). Same conventions as in (a). Each pareidolia image had a matched non-pareidolic control image depicting the same kind of object (e.g., a chair or a faucet; Fig. 1a). c, d. Scatterplot showing the correlation between face selectivity (face d') plotted on the x-axis and scrambled object rank correlation on the y-axis for CIT (c) and AIT (d). Same conventions as in a, b. To evaluate whether the selectivity profile across various objects was the same for pareidolia and control images, we calculated an object rank correlation for each unit. This metric was defined as the Spearman correlation between the response vector to all pareidolia images and the response vector to all corresponding controls. A positive object rank correlation indicates that a neural unit responds to features shared between pareidolia images and their matched controls, but distinct across object identities (e.g., properties like texture or color that are associated with object identity in our stimulus set). Conversely, an object rank correlation of zero indicates that the unit did not selectively respond to features differentiating between object identities. We found a significant positive object rank correlation in face units (CIT: 0.2 ± 0.1,

t_{112} = 21.43, p = 2 x 10^{-41}, AIT = 0.04 ± 0.1, t_{121} = 3.86, p = 1.82 x 10^{-4}) and in non-face units (CIT: 0.25 ± 0.12, t_{94} = 20.5, p = 1.93 x 10^{-36}, AIT = 0.27 ± 0.1, t_{40} = 15.67, p = 1.14 x 10^{-18}). Further, the object rank correlation for face units was significantly lower than for nonface units, particularly in AIT (CIT: t_{206} = -3.23, p = 0.0014, AIT: t_{161} = -12.44, p = 2.46 x 10^{-25}). This effect is explained by the negative correlation between face selectivity and object rank correlation (CIT: n = 208, Pearson's r = -0.23, p = 0.00083, AIT: n = 163, Pearson's r = -0.65, p = 6.26 x 10^{-21}). This result suggests that the more face selective units in CIT and AIT were driven less by object features that differentiate between object identities, and more by object features shared among actual and illusory faces. However, our results also indicate that this may not be the face-like configuration since scrambling the configuration preserves the negative correlation between face selectivity and object rank correlation (CIT: original r = -0.23, scrambled r = -0.3, difference in r=-0.07, 95%CI[-0.22,0.07]; AIT: original r = -0.65, scrambled r = -0.41, difference in r=0.24, 95%CI[0.11,0.37]).

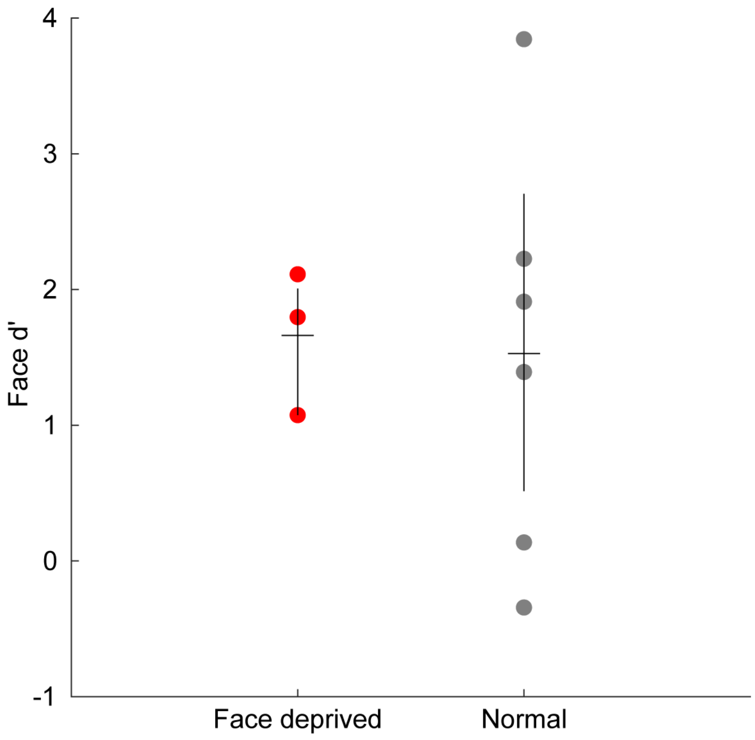

Supplementary Fig. 5. Face deprived and normal monkeys do not differ in face selectivity. Scatterplot showing the face selectivity (quantified by the metric face d') on the x-axis for face-deprived (red circles) and control monkeys (gray circles). The horizontal line shows the mean face d' for each group, vertical lines depict the confidence intervals. The difference between face d' for the face deprived monkeys was not significantly different from the control monkeys (t_7 = 0.08, p = 0.94).

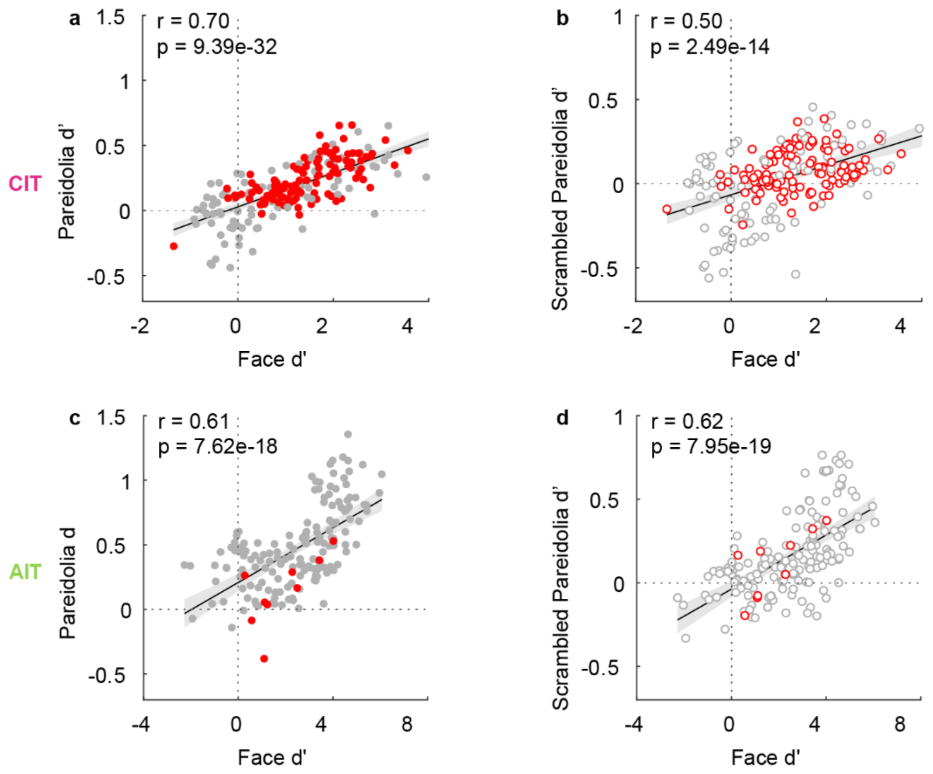

Supplementary Fig. 6. Pareidolia selectivity of neural units in face deprived monkeys falls within the range of normal monkeys. Scatterplot showing the face selectivity on the x-axis and pareidolia selectivity on the y-axis in face-deprived (red circles) and control monkeys (gray circles) for original and scrambled pareidolia images in CIT (a, b) and AIT (c, d). The black line indicates an ordinary least squares (OLS) linear regression fit, with shaded 95% confidence intervals bands. The values on the top left corner depict the Pearson's correlation and the corresponding p-value calculated using the corr function in MATLAB (R2021b).

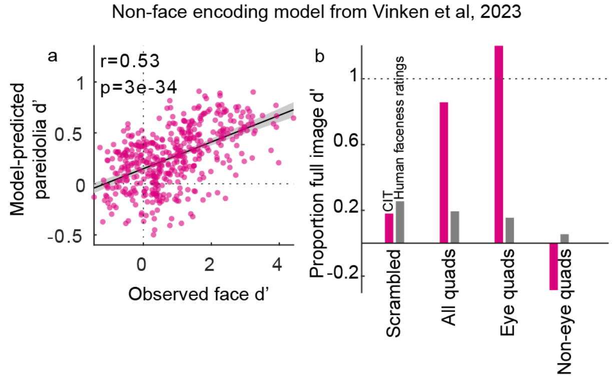

Supplementary Fig. 7. Pareidolia selectivity is predicted by the feature-tuning estimated from non-face objects. a. Scatterplot showing the correlation between observed neural face selectivity and model-predicted pareidolia selectivity, using the independent non-face encoding model from Vinken and colleagues[1]. This independent model was constructed using a CNN-based encoding model (using the same base model as above) and was fit (linearly mapped) on the responses from 449 central IT sites to 932 exclusively inanimate, non-face objects (for more details see Methods and [1]). The black line indicates an ordinary least squares (OLS)

linear regression fit, with shaded 95% confidence intervals bands. The values on the top left corner depict the Pearson's correlation and the corresponding p-value calculated using the corr function in MATLAB (R2021b). b. The proportion of mean d' value (relative to the d' of the full original images) for quadrant scrambled, all quadrants, eye quadrants only and non-eye quadrants only, averaged across 190 face units (face d'>1) in central IT (CIT; pink bar) and 100 human subjects' faceness ratings (gray bar) for a subset of 34 images.

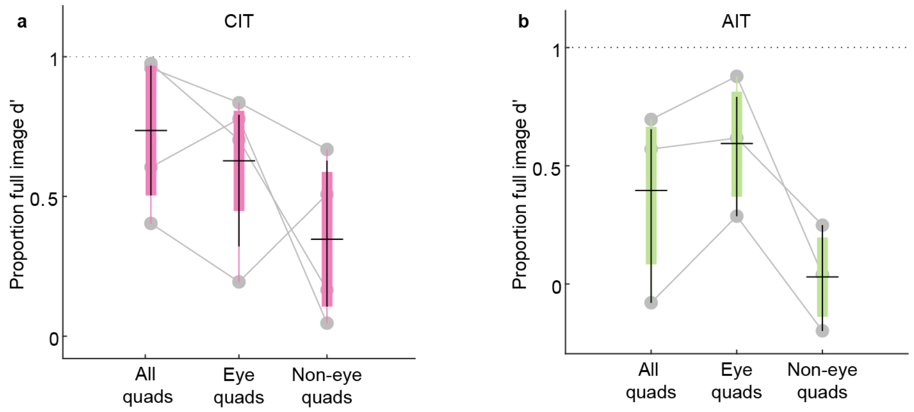

Supplementary Fig. 8. The contribution of isolated quadrans in driving the face-cell pareidolia selectivity per monkey. a. The proportion of mean d' value (relative to the d' of the full original images) for all quadrants, eye quadrants only and non-eye quadrants only for central IT (CIT) of 4 monkeys is shown in box plots. The black central horizontal line shows the mean across monkeys, the black central vertical line indicates confidence intervals, and the bottom and top edges of the box depict the 25th and 75th percentiles, respectively. Whiskers (vertical lines in color) show the most extreme data points not considered outliers. Grey circles connected by grey lines behind the box plots show the proportion mean d' per monkey. b. The proportion of mean d' value (relative to the d' of the full original images) for all quadrants, eye quadrants only and non-eye quadrants only for anterior IT (AIT) of 3 monkeys. Same conventions as in a. Note that in this figure, the proportion of the mean d' was calculated individually for each monkey based on that monkey's average pareidolia d' for all quadrants, eye quadrants only, non-eye quadrants only and the full pareidolia images.

Monkey ID (no. of units)	CIT				AIT			
	Original		Scrambled		Original		Scrambled	
	r	p	r	p	r	p	r	p
M1 (64)	0.53	8.4×10^{-6}	0.13	0.3				
M2 (FD; 73)	0.63	1.9×10^{-9}	0.45	7.1×10^{-5}				
M3 (31)	0.4	0.027	0.27	0.15				
M4 (FD; CIT: 40; AIT: 9)	0.24	0.14	0.04	0.83	0.69	0.042	0.74	0.024
M5 (60)					0.44	0.0004	0.32	0.013
M6 (13)					0.74	0.0036	0.4	0.18
M7 (24)					0.58	0.0028	0.71	0.0001
M8 (57)					0.53	1.9×10^{-5}	0.4	0.0028
Mean	*0.45*		*0.22*		*0.6*		*0.51*	

Supplementary Table 1. Correlation between pareidolia and face selectivity per monkey. Table showing Pearson's correlation *r* between pareidolia d' and face d' for each monkey in central (CIT; n = 4) and anterior IT (AIT; n = 5) for original and quadrant scrambled images from Experiment 1. One-sample, one-tailed t-tests on the Fischer-transformed correlation values indicate that this correlation was significant across monkeys for both original and scrambled images (Original - CIT: $t_3 = 4.62$, p = 0.0096; AIT: $t_4 = 8.19$, p = 0.00061; Scrambled - CIT: $t_3 = 2.4$, p = 0.049; AIT: $t_4 = 4.61$, p = 0.005). FD = face deprived.

Monkey ID (no. of units)	CIT		AIT	
	r	p	r	p
M1 (41)	0.54	0.0003		
M2 (FD; 52)	0.034	0.81		
M3 (27)	0.46	0.017		
M4 (FD; 25)	0.81	1.23×10^{-6}		
M5 (10)			0.49	0.15
M6 (12)			0.76	0.0042
M7 (1)			n/a	n/a
M8 (57)			0.72	4.9×10^{-9}
Mean	*0.46*		*0.66*	

Supplementary Table 2. Correlation between pareidolia selectivity for eye parts only and full pareidolia images. Table showing Pearson's correlation *r* between pareidolia d' for pareidolia eye parts only and full pareidolia images from Experiment 4 for each monkey in central (CIT; n = 4) and anterior IT (AIT; n = 3). One-sample, one-tailed t-tests on the Fischer-transformed correlation values indicate that this correlation was significant across monkeys (CIT: $t_3 = 2.52$, p = 0.043; AIT: $t_2 = 5.8$, p = 0.014). FD = face deprived.

References

1. Vinken, K., Prince, J. S., Konkle, T. & Livingstone, M. S. The neural code for "face cells" is not face-specific. *Sci Adv* **9**, (2023).
