# Supplementary material for: Face cells encode object parts more than facial configuration of illusory faces
Source: Nat Commun. 2024 Nov 14;15:9879. doi: 10.1038/s41467-024-54323-w (PMC11564726; doi:10.1038/s41467-024-54323-w)
Supplement: Supplementary file 2 — Reporting Summary [file 41467_2024_54323_MOESM2_ESM.pdf]

nature portfolio

Corresponding author(s): Saloni Sharma

Last updated by author(s): Oct 15, 2024

Reporting Summary

Nature Portfolio wishes to improve the reproducibility of the work that we publish. This form provides structure for consistency and transparency in reporting. For further information on Nature Portfolio policies, see our Editorial Policies and the Editorial Policy Checklist.

Statistics

For all statistical analyses, confirm that the following items are present in the figure legend, table legend, main text, or Methods section.

n/a	Confirmed	
☐	☒	The exact sample size (n) for each experimental group/condition, given as a discrete number and unit of measurement
☐	☒	A statement on whether measurements were taken from distinct samples or whether the same sample was measured repeatedly
☐	☒	The statistical test(s) used AND whether they are one- or two-sided<br>*Only common tests should be described solely by name; describe more complex techniques in the Methods section.*
☐	☒	A description of all covariates tested
☐	☒	A description of any assumptions or corrections, such as tests of normality and adjustment for multiple comparisons
☐	☒	A full description of the statistical parameters including central tendency (e.g. means) or other basic estimates (e.g. regression coefficient) AND variation (e.g. standard deviation) or associated estimates of uncertainty (e.g. confidence intervals)
☐	☒	For null hypothesis testing, the test statistic (e.g. F, t, r) with confidence intervals, effect sizes, degrees of freedom and P value noted<br>*Give P values as exact values whenever suitable.*
☒	☐	For Bayesian analysis, information on the choice of priors and Markov chain Monte Carlo settings
☐	☒	For hierarchical and complex designs, identification of the appropriate level for tests and full reporting of outcomes
☐	☒	Estimates of effect sizes (e.g. Cohen's d, Pearson's r), indicating how they were calculated

Our web collection on statistics for biologists contains articles on many of the points above.

Software and code

Policy information about availability of computer code

Data collection	Neural recordings were performed on a Plexon Omniplex acquisition system and neural data were collected using proprietary software (Omniplex, Plexon, Dallas, TX). Experiments were controlled using the NIMH MonkeyLogic MATLAB toolbox, which is an open-source software (available at https://monkeylogic.nimh.nih.gov). For the human behavioral study, the participants were recruited from an online website Prolific (https://www.prolific.com/) and then redirected to our own website where we used jsPsych, a JavaScript-based framework, to run the experiment.
Data analysis	The data were analyzed using custom MATLAB code (R2021b). The custom-written code necessary to reproduce the data figures in this paper are publicly available in the OSF database (https://osf.io/z2kra/).

For manuscripts utilizing custom algorithms or software that are central to the research but not yet described in published literature, software must be made available to editors and reviewers. We strongly encourage code deposition in a community repository (e.g. GitHub). See the Nature Portfolio guidelines for submitting code & software for further information.

Data

Policy information about availability of data

All manuscripts must include a data availability statement. This statement should provide the following information, where applicable:

- Accession codes, unique identifiers, or web links for publicly available datasets
- A description of any restrictions on data availability
- For clinical datasets or third party data, please ensure that the statement adheres to our policy

Data generated and used in this study have been deposited on the Open Science Framework (OSF) database (https://osf.io/z2kra/). The data are available under full access, access can be obtained by downloading the .mat files. All source data necessary to preproduce the figures in this paper are publicly available in the OSF database (https://osf.io/z2kra/). Source data are provided with this paper.

Research involving human participants, their data, or biological material

Policy information about studies with human participants or human data. See also policy information about sex, gender (identity/presentation), and sexual orientation and race, ethnicity and racism.

Reporting on sex and gender	Of the 100 participants included in the final analysis, 50 self-identified as male, 48 as female, and 2 gave no response to the gender question. We did not consider sex/gender in the study design and included all 100 participants in our analysis.
Reporting on race, ethnicity, or other socially relevant groupings	Of the 100 participants included in the final analysis, 56 self-identified as White, 19 as Back, 13 as Mixed, 2 as Asian, 5 as Other, and 5 gave no response to this question. We did not take race into consideration in the study design and included all 100 participants in our analysis.
Population characteristics	A total of 100 adults (mean age = 31.8 y, SD = 10.7) participated in the experiments via the online crowdsourcing platform Prolific.
Recruitment	A total of 100 human subjects were randomly recruited from an online platform Prolific without any specific selection critera. The participants were then redirected to our own website where we used jsPsych, a JavaScript-based framework, to run the experiment. All participants provided informed consent and received monetary compensation for participation in the experiment.
Ethics oversight	The experiment was conducted according to protocols approved by the Institutional Review Board at Harvard Medical School.

Note that full information on the approval of the study protocol must also be provided in the manuscript.

Field-specific reporting

Please select the one below that is the best fit for your research. If you are not sure, read the appropriate sections before making your selection.

☒ Life sciences ☐ Behavioural & social sciences ☐ Ecological, evolutionary & environmental sciences

For a reference copy of the document with all sections, see nature.com/documents/nr-reporting-summary-flat.pdf

Life sciences study design

All studies must disclose on these points even when the disclosure is negative.

Sample size	The sample size was not predetermined. We included all 8 monkeys with working IT arrays in all our analysis, which exceeds the two-monkey standard in the field. We clearly indicate the sample size in each of our analysis. For the human behavioral study, we included 100 subjects to ensure that we had enough repeats for each unique trial type. All 100 subjects were included in our analysis, which is indicated in the manuscript.
Data exclusions	We did not exclude any experimental sessions or monkeys from our analysis. For the neural units, we only included units that demonstrated reliable visually evoked responses, which was determined by sites demonstrating a split-half reliability of responses across all images higher than 0.4. This cut-off was determined on the basis of previous experiments in the lab. For the human behavioral study, we also did not exclude any participants since all 100 participants completed the experiment.
Replication	Neural results were replicated over all 8 animals, behavioral data was replicated across 100 human subjects
Randomization	No experimental groups were involved in this study
Blinding	Blinding was not necessary since no experimental groups were involved in this study

Reporting for specific materials, systems and methods

We require information from authors about some types of materials, experimental systems and methods used in many studies. Here, indicate whether each material, system or method listed is relevant to your study. If you are not sure if a list item applies to your research, read the appropriate section before selecting a response.

Materials & experimental systems

n/a	Involved in the study
☒	☐ Antibodies
☒	☐ Eukaryotic cell lines
☒	☐ Palaeontology and archaeology
☐	☒ Animals and other organisms
☒	☐ Clinical data
☒	☐ Dual use research of concern
☒	☐ Plants

Methods

n/a	Involved in the study
☒	☐ ChIP-seq
☒	☐ Flow cytometry
☒	☐ MRI-based neuroimaging

Animals and other research organisms

Policy information about studies involving animals; ARRIVE guidelines recommended for reporting animal research, and Sex and Gender in Research

Laboratory animals	Seven adult male Macaca mulatta (5-13 kg; 2-17 years old) and one adult male Macaca nemestrina (15kg, 15 years old) were used in this study.
Wild animals	The study did not involve wild animals.
Reporting on sex	Seven adult male Macaca mulatta (5-13 kg; 2-17 years old) and one adult male Macaca nemestrina (15kg, 15 years old) were used in this study. Our findings are only applicable to males, since we only recorded from male monkeys due to availability.
Field-collected samples	The study did not involve field-collected samples.
Ethics oversight	All procedures were approved by the Harvard Medical School Institutional Animal Care and Use Committee and conformed to NIH guidelines provided in the Guide for the Care and Use of Laboratory Animals.

Note that full information on the approval of the study protocol must also be provided in the manuscript.

Plants

Seed stocks	*Report on the source of all seed stocks or other plant material used. If applicable, state the seed stock centre and catalogue number. If plant specimens were collected from the field, describe the collection location, date and sampling procedures.*
Novel plant genotypes	*Describe the methods by which all novel plant genotypes were produced. This includes those generated by transgenic approaches, gene editing, chemical/radiation-based mutagenesis and hybridization. For transgenic lines, describe the transformation method, the number of independent lines analyzed and the generation upon which experiments were performed. For gene-edited lines, describe the editor used, the endogenous sequence targeted for editing, the targeting guide RNA sequence (if applicable) and how the editor was applied.*
Authentication	*Describe any authentication procedures for each seed stock used or novel genotype generated. Describe any experiments used to assess the effect of a mutation and, where applicable, how potential secondary effects (e.g. second site T-DNA insertions, mosiacism, off-target gene editing) were examined.*
